# Understanding the role of aerobic fitness, spatial learning, and hippocampal subfields in adolescent males

**DOI:** 10.1038/s41598-021-88452-9

**Published:** 2021-04-29

**Authors:** Sandhya Prathap, Bonnie J. Nagel, Megan M. Herting

**Affiliations:** 1 Department of Preventive Medicine, University of Southern California, Los Angeles, CA 90023 USA; 2 Neuroscience Graduate Program, University of Southern California, Los Angeles, CA 90023 USA; 3 Departments of Psychiatry and Behavioral Neuroscience, Oregon Health and Science University, Portland, OR 97239 USA

**Keywords:** Cognitive neuroscience, Hippocampus

## Abstract

Physical exercise during adolescence, a critical developmental window, can facilitate neurogenesis in the dentate gyrus and astrogliogenesis in Cornu Ammonis (CA) hippocampal subfields of rats, and which have been associated with improved hippocampal dependent memory performance. Recent translational studies in humans also suggest that aerobic fitness is associated with hippocampal volume and better spatial memory during adolescence. However, associations between fitness, hippocampal subfield morphology, and learning capabilities in human adolescents remain largely unknown. Employing a translational study design in 34 adolescent males, we explored the relationship between aerobic fitness, hippocampal subfield volumes, and both spatial and verbal memory. Aerobic fitness, assessed by peak oxygen utilization on a high-intensity exercise test (VO_2_ peak), was positively associated with the volumetric enlargement of the hippocampal head, and the CA1 head region specifically. Larger CA1 volumes were also associated with spatial learning on a Virtual Morris Water Maze task and verbal learning on the Rey Auditory Verbal Learning Test, but not recall memory. In line with previous animal work, the current findings lend support for the long-axis specialization of the hippocampus in the areas of exercise and learning during adolescence.

## Introduction

Low physical activity levels during adolescence is a growing public health concern^1,2^, with less than 8% of adolescents meeting the daily exercise requirements of 1 hour of physical activity^3^ recommended by World Health Organization and the US Department of Health^4–6^. Adolescence is a key transitional period of physical, emotional, and social growth^7^ characterized by substantial brain growth^8–10^. Rapid neural maturation and concomitant delayed cognitive development during this critical period of development makes this population especially sensitive to environmental and lifestyle influences, such as physical exercise.

Aerobic fitness can be defined as the body’s ability or capacity to engage the respiratory and circulatory systems to deliver oxygen during an activity^11^. Aerobic fitness and more generally exercise is linked to improved learning and memory abilities during adolescence^12–15^, and may act as a protective factor against physical and mental health problems later in life^16–18^. Animal studies have established that voluntary exercise increases neurogenesis in the dentate gyrus (DG)^19^. In addition, exercise has been linked to differences in cell density as determined by Nissl stain Cornu Ammonis areas CA1 and CA3 regions of the hippocampus during adolescence in juvenile male rats^20^. Subsequent research in male rats also suggests exercise-related astrocytic changes within the hippocampus^21–23^, including increases in astrocyte density and morphology in the CA1 region^22^. Furthermore, these exercise-induced changes occur in conjunction with enhanced hippocampal dependent memory abilities, such as spatial memory performance in the Morris Water Maze task, a powerful tool for assessing spatial mapping abilities in rodents^19,20^. Translating these studies to humans, our previous work has utilized peak oxygen consumption testing (i.e., VO_2_ peak; the gold-standard in assessing aerobic fitness), a virtual Morris Water Maze paradigm, and a cross-sectional design to establish that aerobic fitness is associated with larger hippocampal volumes and superior spatial learning abilities on a virtual Morris Water Maze in adolescent males^24^. Based on prior animal literature^20,22^, aerobic exercise is likely to have regional effects on hippocampal morphology; however, this has been understudied in an adolescent population^14,25^. Previous imaging techniques were initially limited to global volumetric analysis of the hippocampus, which likely masks specificity of anatomical changes within structurally and functionally distinct hippocampal subfields. However, recent advances utilizing histologically-validated parcellations of hippocampal subfields^26^ allow for further exploration of regional specificity regarding the translation of exercise and learning associations with hippocampal morphology from animal to human studies. Cross-species comparisons of hippocampal subfields between rats and humans are vital to better inform our understanding of both macro and micro scale hippocampal plasticity induced by exercise, and whether structural morphology relates to spatial learning and memory abilities during adolescence.

Cross-sectional studies in children^27,28^, adolescents^24^, and older adults^29^ reveal that higher fitness levels are associated with larger hippocampal volumes and superior cognitive performance. Human structural and functional magnetic resonance imaging (MRI) research over the past decade suggests that exercise and various memory abilities may differentially affect specific subfields within the hippocampus. Randomized control trials (RCTs) in both young and older adults have also localized aerobic exercise training improvements with the enlargement of cortical grey matter^30–32^, changes in neurovasculature^33,34^, resting state functional connectivity^35^, and myelination^31^ within the anterior portion of the hippocampus. The anterior hippocampus (ventral hippocampus in rats) is a neurogenic zone^36–38^. Various aspects of spatial navigation (e.g. encoding vs. retrieval) are also thought to be functionally differentiated along the longitudinal axis of the hippocampus^39,40^. Other types of memory such as verbal learning and delay recall, a type of episodic memory, is also thought to involve the anterior hippocampus^41^. Within the anterior region, a few RCTs have further localized exercise-induced sMRI growth and increased cerebral blood volume to the DG region^32,34^ (i.e. the site of neurogenesis which creates adult granule cells^36,37^) and CA3^32^. On the other hand, others have only observed sMRI increases in the CA1 subfield following a RCT intervention^42^. Across the hippocampal longitudinal axis, the DG and CA regions are both crucial for spatial navigation^43^; however, the DG is responsible for pattern separation^44,45^; CA3 for pattern completion^46,47^; and CA1 is more generally involved with encoding^48,49^. While these MRI studies suggest that the DG, CA1, and CA3 subfields are influenced by exercise, there is a lack of consistency with respect to MRI resolution and hippocampal segmentation methods, and age group—which primarily focuses on older adult populations. Moreover, few studies have looked to examine how exercise and learning behaviors both map to hippocampal subfields during adolescent development.

The neuroprotective effects of exercise during the period of adolescence has been largely understudied^14,25^. Adolescence presents a unique phase of development in which neural circuitry and cognitive functions have not reached peak development. SMRI studies reveal that overall hippocampal volume^50,51^ and hippocampal subfield volumes follow a protracted developmental trajectory; peaking during early adolescence^52^. Furthermore, exercise may differentially modify hippocampal plasticity during the ongoing development of these systems during adolescence, as more robust hippocampal neurogenesis is observed in young adult rats compared to older adult rats^53^. Altogether, exercise may hold great promise to enhance learning and memory capabilities during the teenage years—which is a critical period for academic achievement^12,13^. Identifying subfield structural correlates associated with aerobic fitness, spatial, and verbal memory domains during this developmental period is necessary to localize exercise induced hippocampal plasticity within subfields.

**Figure 1:**
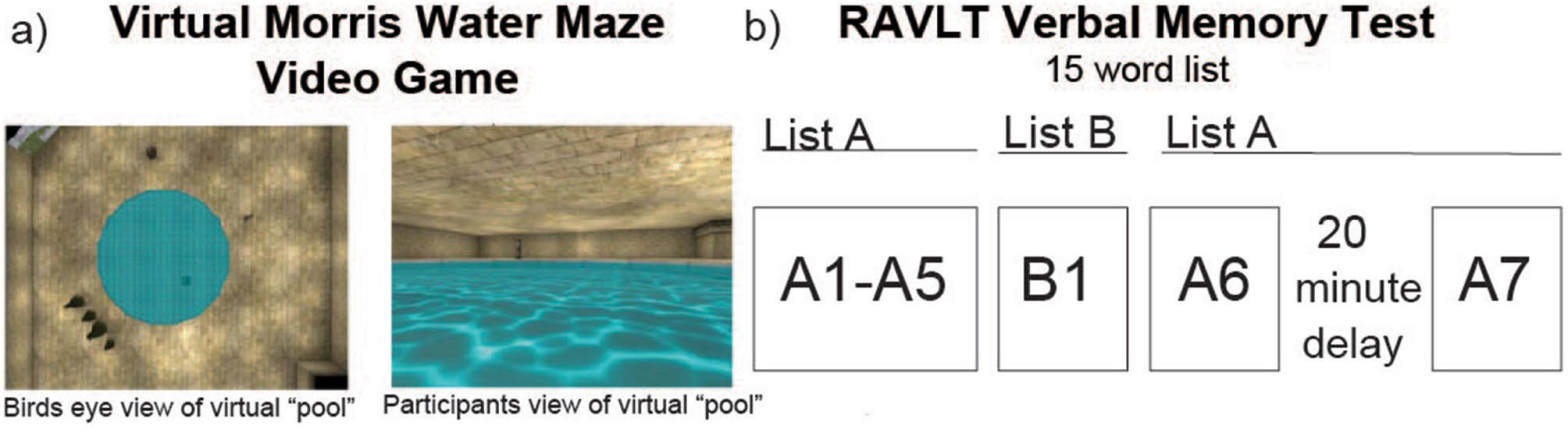
Spatial and verbal memory tasks. (**a**) Snapshots of the virtual Morris Water maze task^57^, including a view of the virtual pool the participant navigates in. Similar to the animal Morris Water Maze Task, participants were asked to navigate through the virtual pool using a joystick to find a hidden platform. Permission to use screenshots from this task was granted by Dr. Moffat^57^. (**b**) Sequence of the RAVLT memory task. Participants were asked to learn a list of 15 words and were later tested on number of words remembered after a delay of 20 min. Participants initially learned 15 words from list A and B, but were only tested on list A.

Employing a translational approach, the aim of the present study was to determine if aerobic fitness and virtual Morris Water Maze Task performance (vMWT) (Fig. 1a) are associated with specific T1-weighted MRI structural subfield volumes of the hippocampus in 34 adolescent males. Importantly, all adolescents were of healthy (i.e. non-obese) weight status; reducing body weight effects which can possibly confound studies trying to capture ‘fitness’ effects^14^. Linear mixed effect models were used to compute associations between aerobic fitness, spatial learning and memory performance, and hippocampal subfields for each hemisphere. Finally, associations between a verbal learning and memory task (i.e. Rey Auditory Verbal Learning Test (RAVLT); (Fig. 1b) and hippocampal subfields were also assessed to probe potential specificity between the spatial and verbal learning and memory domains. Based on findings from the animal literature^20,22^, we predicted that higher aerobic fitness levels would be associated with larger subfield volumes in the head region, specifically within the DG, CA1, and CA3 head subfields. Given previous human hippocampal subfield studies in the domains of spatial^49,54^ and episodic verbal memory^41^, we also expected spatial learning to be associated with larger subfield volumes in the DG and CA1 subregion, and both verbal learning and memory to be associated with subfield volumes in the head region.

## Results

### Aerobic fitness is positively related to CA1 head subfield

**Figure 2:**
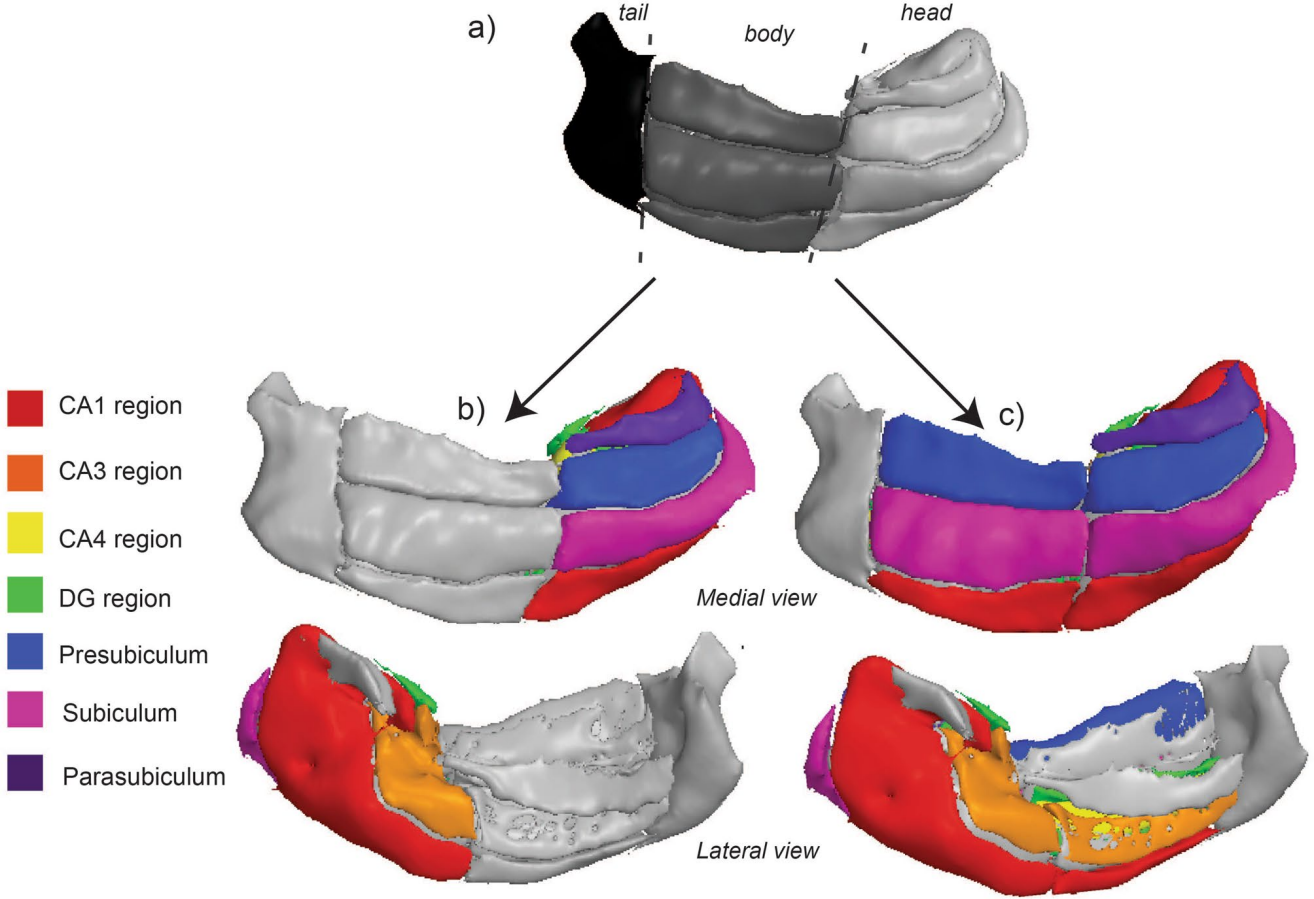
Visual representation of anterior to posterior longitudinal axis and subfield model building strategy. (**a**) We first examined a potential interaction between the independent variables (IVs) of interest and regional differences in volume along the longitudinal axis (head, body, tail ROIs) of the hippocampus using a linear mixed model analysis. (**b**) If interaction of the initial model was significant, follow-up analyses examined volume differences of the subfields of interest (i.e. CA1, CA3, CA4, DG, presubiculum, subiculum, and parasubiculum) within the longitudinal axis region identified by the initial model. A priori animal and human literature determined the specific subfields that were in included in the analysis for each IV of interest. (**c**) If no differences were observed in volume along the longitudinal axis (i.e. interaction term for the longitudinal axis was not significant), then the subfields of interest (i.e. CA1, CA3, CA4, DG, presubiculum, subiculum, and parasubiculum) were examined for the entire length of the hippocampal formation. Hippocampal images were created using Quantitative Imaging Toolkit (QIT)^143^.

**Figure 3:**
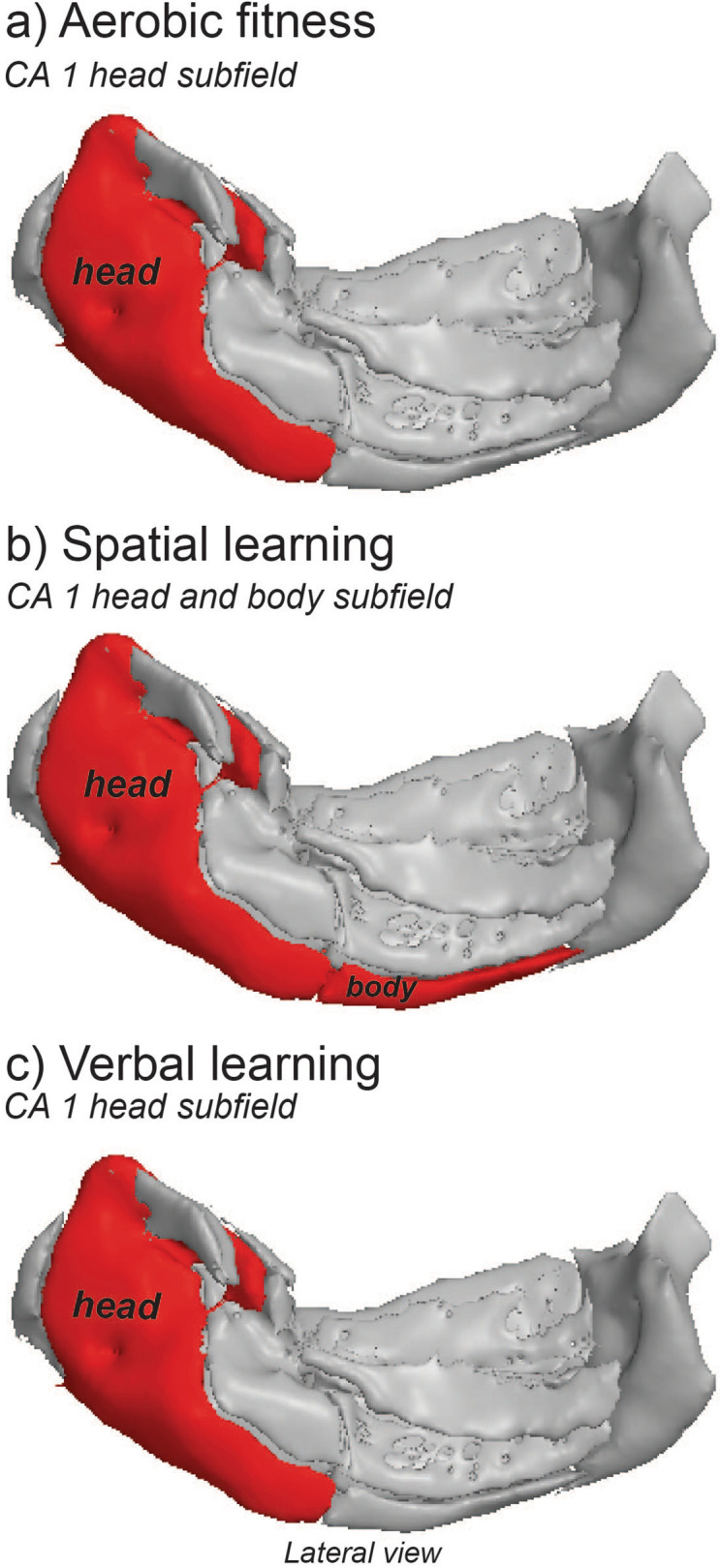
Hippocampal subfields associated with exercise and cognitive domains. A 3-D representation of the positive associations between IV variables of interest: (**a**) aerobic fitness, (**b**) spatial learning, and (**c**) verbal learning with CA1 subfields. Positive associations correspond to larger volumes within the CA1 subfield. Hippocampal images were created using Quantitative Imaging Toolkit (QIT)^143^.

Participants completed the gold-standard Bruce protocol test^55^ for quantifying aerobic fitness by peak oxygen consumption (VO_2_ peak, adjusted for lean body mass (ml/kg LBM/min))^56^ and structural 3T T1-weighted images (Table 1). Based on the literature, an a priori model building strategy was used to examine the association between VO_2_ peak and hippocampal subfields along the anterior–posterior axis of the hippocampus (Fig. 2a). First, we examined a potential interaction between VO_2_ peak (ml/kg LBM/min) and regional differences in volume along the longitudinal axis, including the head, body, and tail of the hippocampus (Fig. 1a). Hippocampus volumes significantly differed along the longitudinal axis in relation to aerobic fitness (F(2,165) = 5.78, *p* = 0.004, R^2^ model = 0.96). Follow-up analyses revealed a significant association between aerobic fitness and hippocampal volume in the head, whereas this association was not significant in the body or tail (Table 2). Next, we performed follow-up analyses to probe if aerobic fitness related to volumetric differences of the subfields within the head region of the longitudinal axis of the hippocampus (Fig. 2b). Given previous findings from animal literature^20,22^, we examined the CA1, CA3, CA4, and DG subfields. Scatter plot of VO_2_ peak with the hippocampus head, as well as each of the head subfield volumes are presented in Supplementary Fig. S2. In final models taking into consideration the shared variance of subfield volumes between and within hemispheres for any given individual, and adjusting for covariates, significant differences were observed with aerobic fitness and distinct subfield volumes (F(3,231) = 12.58, *p* = 0.001, R^2^ model = 0.98), which was driven by a positive association between aerobic fitness and the CA1 head region (Table 2, Fig. 3). The Shapiro-Wilks test confirmed that the relevant variables did not violate assumptions of normality (see Supplementary Table S1).

**Table 1:** Participant demographics.

	Mean	sd	n
**Demographics**			
Age	16.42	0.82	34
PDS	3.19	0.39	34
IQ	117.56	9.62	34
SES	22.41	10.75	34
Household income	128.24	73.01	33
BMI	22	3.38	34
VO_2_ peak (ml/kg/min)	63.76	11.93	34
LBM based VO_2_ peak (ml/kg LBM/min)	72.35	10.46	34
vMWT Spatial learning	0.35	0.22	30
vMWT Spatial delay recall	2	1.49	30
RAVLT Verbal learning (z score)	1.73	0.82	33
RAVLT Verbal delay recall	− 0.39	1.58	33
MRI volumes* (mm^**3**^**)**			
Hippocampus	4034.51	472.06	34
Hippocampal head	2095.40	245.22	34
Hippocampal body	1388.87	152.12	34
Hippocampal tail	637.56	69.81	34
CA1 head	635.83	77	34
CA3 head	162.17	25.63	34
CA4 head	162.88	22.61	34
DG head	197.01	28.18	34
Presubiculum head	165.84	21.53	34
Subiculum head	213.91	30.92	34
CA1 body	145.75	27.64	34
CA3 body	99.15	19.46	34
CA4 body	139.94	18.61	34
DG body	158.66	21.31	34
Presubiculum body	193.14	24.59	34
Subiculum body	281.94	30.20	34
Parasubiculum	81.41	13.33	34

*sd* standard deviation, *IQ* Wechsler Abbreviated Scale of Intelligence, *SES* Hollingshead Index of Social position, *vMWT* Virtual Morris Water Maze Task, *RAVLT* Rey Auditory Verbal Learning Test, *CA* cornu ammus, *DG* dentate gyrus.

*Left and right region of each subfield are combined.

**Table 2:** Association between VO_2_ peak and hippocampal volumes.

VO_2_ peak	df	Beta	se	t-stat	*p* value	R^2^ partial
**Longitudinal axis volumes**						
Head**	30	6.95	2.34	2.97	**0.006**	0.04
Body	30	2.45	2.34	1.05	0.31	0.002
Tail	30	1.32	2.34	0.56	0.58	0.005
**Head subfield volumes**						
CA1**	30	2.56	0.6	4.26	**0.0002**	0.2
CA3	30	0.11	0.6	0.18	0.86	0.168
CA4	30	0.33	0.6	0.56	0.56	0.088
DG	30	0.49	0.6	0.082	0.42	0.075

Table of the associations between VO_2_ peak and hippocampal subfields using linear mixed effect models.

**p < 0.007.

Bold text reflects significant *p*-values after Bonferroni correction.

### Spatial learning on the Virtual Morris Water Maze Task relates to CA1 subfield volume

In order to improve translation of findings between animal and human studies, spatial learning and memory were assessed using a computerized virtual Morris Water Maze task (vMWT) aimed to mirror the animal version of the task^57^ (Fig. 1a). Participants used a joystick to “swim” and find a hidden platform in a computerized environment of a pool set inside a larger room. Within the pool there was a hidden platform, and the task required participants to learn the pool environment, using a set of spatial cues, to find the hidden platform as fast as possible. When the participant swam over the hidden platform, it emerged from the water and hoisted the participant out of the pool for 10 seconds (s) indicating that a trial was completed. Following a practice trial, the individual completed 6 learning trials. During the learning trials, participants were told that the hidden platform would remain in the same new position for all of the trials. While the participant searched for and found the hidden platform, the computer recorded the (x, y) coordinate position approximately every 0.02s. Using this method, the computer calculated the total distance traveled in each quadrant of the pool, including the quadrant that the hidden platform was located in—known as the target quadrant. Percent distance traveled (i.e. total distance traveled in the target quadrant divided by the total distance traveled across all quadrants) was calculated for each of the 6 learning trials. The amount of learning that occurred over the 6 trials was captured by improvements in the percent of time, or delta (δ), from the first to last learning trial (δ = percent distance trial 6 − percent distance trial 1). We found that spatial learning on the vMWT was not associated with volumetric differences along the longitudinal axis (i.e. head, body, and tail segments; F(2,145) = 2.13, *p* = 0.13, R^2^ model = 0.96). Animal studies suggest that hippocampal place cells, located within the subiculum-related and CA subfields, are integral for spatial memory formation^58^. Based on this rationale, we then further examined whether spatial learning related to volumes within the subfields of the CA1, CA3, CA4, DG, parasubiculum, presubiculum, and subiculum (Fig. 2c). The association between spatial learning and volumes across these 7 subfields were significantly different (F(6,377) = 3.21, *p* = 0.005, R^2^ model = 0.97). Larger CA1 volumes were found to relate to better spatial learning across the 6 trials of the task (Table 3, Fig. 3b).

**Table 3:** Association between spatial and verbal learning on hippocampal volumes.

	df	Beta	se	t-stat	*p* value	R^2^ partial
**Spatial learning**						
Subfield volumes						
CA1**	26	122.6	37.21	3.29	**0.003**	0.07
CA3	26	20.18	37.21	0.54	0.6	0.03
CA4	26	24.43	37.21	0.66	0.52	0.03
DG	26	31.27	37.21	0.84	0.41	0.03
Parasubiculum	26	8.52	37.21	0.23	0.83	0.04
Presubiculum	26	26.24	37.21	0.71	0.49	0.03
Subiculum	26	47.61	37.21	1.28	0.22	0.02
**Verbal learning**						
Longitudinal axis volumes						
Head**	29	101.28	28.38	3.57	**0.002**	0.07
Body	29	35.59	28.38	1.25	0.22	0.002
Tail	29	9.47	28.38	0.33	0.75	0.006
Head subfield volumes						
CA1**	29	27.39	6.47	4.23	**0.001**	0.11
CA3	29	9.67	6.47	1.49	0.15	0.03
CA4	29	9	6.47	1.39	0.18	0.03
DG	29	11.79	6.47	1.82	0.08	0.02
Presubiculum	29	7.95	6.47	1.23	0.23	0.03
Subiculum	29	10.97	6.47	1.69	0.11	0.02

Table of the associations between spatial and verbal learning and hippocampal subfields using linear mixed effect models.

**p < 0.007.

Bold text reflects significant *p*-values after Bonferroni correction.

30 minutes after the completion of the learning trials on the vMWT, participants were asked to complete a spatial delay recall trial. The spatial delay recall trial was identical to the learning trials, but the hidden platform was removed. This was done to assess whether the participant remembered the location of the hidden platform that was identified during the previous learning trials. Spatial delay recall was calculated as the percent distance traveled in the target quadrant within the first 30s of the trial. Spatial delay recall memory on this task was not associated with volumes along the longitudinal axis of the hippocampus (F(2,145) = 1.31, *p* = 0.28, R^2^ model = 0.96) or any subfield volumes (F(6,377) = 0.67, *p* = 0.69, R^2^ model = 0.97).

Since we had previously shown that aerobic fitness and spatial learning on this task was significant in this sample; (*beta* = 0.012, *p* = 0.001, R^2^ model = 0.34)^24^, a four-step mediation analysis model outlined by Baron and Kinney^59^ was performed to test whether hippocampal subfield volume CA1 would mediate the relationship between aerobic fitness and spatial learning. For this analysis simple linear regression models were utilized, and covariates total ICV and pubertal development were included. Left and right CA1 head and body volumes were summed, and then left and right hemisphere volumes were averaged to create overall CA1 volumes. We found a significant relationship between aerobic fitness and the CA1 subfield (*beta* = 2.96, *p* < 0.04, R^2^ model = 0.38) indicating a significant relationship between the IV and mediator of interest. Given that (1) aerobic fitness predicts both learning on the vMWT, as well as CA1 subfield volumes, and (2) CA1 subfield volumes predicts spatial learning, multiple linear regressions were performed to determine if hippocampal volume predicts spatial learning, while controlling for aerobic fitness. The relationship between the CA1 subfield volume (outcome) and spatial learning (the mediator) (while controlling for the IV of VO_2_ peak) was not significant (*beta* = 0.0003, *p* = 0.45, R^2^ model = 0.38). Thus, the CA1 subfield as a potential mediator does not relate to spatial learning (i.e. the outcome of interest). Since the mediator does not relate to the outcome, conditions were not met to test CA1 subfield volume as a mediator of the relationship between aerobic fitness and spatial learning in the current study. Rather, these findings suggest that the relationship seen between the CA1 subfield and spatial learning is largely driven by individual associations of CA1 subfield volume and spatial learning with aerobic fitness.

### Verbal learning is positively related to CA1 head subfield

Verbal learning was measured using the RAVLT test (Fig. 1b), and a higher verbal learning score reflected a greater number of words learned over 5 learning trials. Verbal learning was found to associate with hippocampus volumes differentially along the longitudinal axis (F(2,160) = 9.0, *p* = 0.001, R^2^ model = 0.96), with larger volumes of the head specifically associated with better verbal learning (Table 3). Again, we performed follow-up analyses to probe if verbal learning related to volumetric differences of the subfields within the head region of the longitudinal axis of the hippocampus. Since a limited number of studies have examined verbal learning with respect to hippocampal subfields^41,49^, all of the head-related CA, DG, and subiculum subfields were included in the follow up analysis. Results showed volumetric differences of the head region in the association with verbal learning (F(5,352) = 3.16, *p* = 0.009, R^2^ model = 0.98), with larger CA1 head volumes associated with learning more words over the 5 learning trials (Table 3, Fig. 3). Performance on the verbal delay recall memory portion of the RAVLT, which asks participants to list all the words they can remember after 20 minutes, was not found to associate with volumes along the longitudinal axis of the hippocampus (F(2,160) = 2.51, *p* = 0.09, R^2^ model = 0.96) or any subfield volumes (F(6,416) = 1.15, *p* = 0.34, R^2^ model = 0.97). In order to test for mediation effects between aerobic fitness, CA1 head volume, and verbal learning, follow-up regression analyses were first performed to determine the relationship between aerobic fitness and verbal learning. There was not a significant association between the two (*p* = 0.618). Therefore associations did not meet criteria for directly testing a mediation analysis^59^.

## Discussion

While studies have demonstrated an association between total hippocampal volumes and aerobic fitness in children and adolescence, this study utilized an advanced hippocampal subfield segmentation technique^26^ to better translate known aerobic exercise and learning associations with hippocampal substructures from animal studies to humans during adolescence. Aerobic fitness, assessed by VO_2_ peak, and verbal learning were found to positively relate to larger hippocampal head volumes, and especially in the CA1 head region. Alternatively, larger CA1 spanning both the head and body of the hippocampus was found to relate to better spatial learning.

In line with our hypothesis, our results revealed that aerobic fitness levels are associated with a larger hippocampal head in adolescent males. Histological evidence dating back to the late 1500 s supports the tripartite division of the hippocampus along the longitudinal axis^60^. Each division displays divergent structural, functional, and gene expression properties^39^. A handful of exercise-based RCT imaging studies in young adult and older populations have identified volumetric increases in both the anterior and head region of the hippocampus associated with fitness improvements^30–32^. Moreover, these results are also in line with rodent exercise studies observing plasticity differences in the ventral hippocampal (anterior equivalent)^61^. The anterior region of the hippocampus displays a high degree of plasticity; it is more suspectable to various environmental and biological processes, such as stress, depression, and aging related neurodegeneration^62–64^. Furthermore, neurogenesis in this region is more easily altered via pharmacological agents^62^. Structurally, the ventral hippocampus uniquely projects to regions associated with cognitive control and emotion regulation (prefrontal cortex, amygdala, nucleus accumbens, and hypothalamic–pituitary–adrenal axis)^65^. Subfields within the anterior hippocampus display less gyrification^66^ and have limited cross-sectional and longitudinal anatomical connectivity to other regions of the hippocampus, as compared to the posterior hippocampus^40^. The limited connectivity within the head region is present in non-human primates, but not observed in rat hippocampal homologue, and may be a signature of higher order species^67^. Interestingly, while some trend level associations were seen between aerobic fitness and volumes of other head subfields, after adjusting for covariates in our final models, we found the association with aerobic fitness was most apparent in the CA1 region within the hippocampal head region. Cellular cytoarchitecture within subfields of the head region display unique morphological properties such as more densely packed pyramidal cell bodies in the CA1 head, and the DG head serving as the primary neurogenic zone, with a larger pool of mature adult granule cells, and a small pool of immature granule neurons^68^. Associations between aerobic fitness and CA1 head volume in the current study are similar to two recent RCT studies which have also localized training induced volumetric increases in gray matter to the enlargement of the CA1 subfield after 12 weeks of aerobic training in young adults^42^, and attenuated CA1 volume atrophy 12 months after cessation of 6 months of resistance training in elderly^69^. The CA1 may play an important role in supplying neurogenic substrates (e.g., glutamate, neurotrophins) to immature granule cells^70^, as the CA1 subfield has heightened upregulation of BDNF, TrkB, and c-fos receptor expression during exercise induced neurogensis^71–74^. Moreover, the current volumetric findings in the CA1 may also be reflective of astrogliogenesis, as physical exercise has been shown to increase astrocyte density as well as morphology in the CA1 region in rodents^22^. While clinical intervention and experimental animal studies provide insight to the neuroplastic effects of exercise over a specific training period, the results of this study indicate that an individual’s overall aerobic fitness may also influence subfield-specific volumes. In addition, similar results between previous studies in young and elderly adults^30–34,42^ and our current findings in an adolescent sample suggest that the localization of exercise-related volumes in the head of the hippocampus may be ubiquitous across the lifespan.

In the current study, spatial and verbal learning performance were also found to be associated with CA1 volumes. Previous literature on the localization of spatial and verbal memory along the longitudinal axis of the hippocampus in humans is mixed. Volumetric and functional studies present discrepant evidence in support of spatial memory localized to both the posterior (body and tail)^75,76^ and anterior (head) hippocampus^77,78^. Our findings suggest that the CA1 region may be important for spatial learning particularly, rather than a specific anterior–posterior division of the hippocampus. The association of larger CA1 volumes and learning on the Morris Water Maze task are in agreement with a recent human lesion study from Bartsch and colleagues^49^. In this study, 14 transient global amnesia patients with focal lesions spanning the entire CA1 region of the hippocampus were found to have significant deficits in spatial learning on a similar virtual Morris Water Maze task, where spatial learning was also defined as the distance traveled across quadrants over a period of time^49^. Similarly, another virtual navigation study associated spatial learning, as defined as passive observation during a navigation task, with fMRI activation of the CA1 region^54^. These results from human studies suggest a role of CA1 in spatial learning, and are further supported by animal experiments which have established that CA1 neurons within the hippocampus are critically involved in both real and virtual world spatial navigation^79–81^. Interestingly, we also found that verbal learning, a type of episodic memory, was found to be positively associated with the volume of the head of the CA1 region. Associations between verbal learning and the head of the hippocampus have been noted in studies of adults. For example, one study in older adults found associations between verbal learning, also assessed by the RAVLT, and larger volumes within in the anterior hippocampus^41^. Functional imaging studies have found that verbal encoding is associated with increased activation in the anterior hippocampus^82^, and specifically the head region of the hippocampus during encoding of verbal stimuli compared with item stimuli^83^. Lastly, patients with focal lesions to the CA1 region (along the head and body), have shown deficits on RAVLT learning^49^. Taken together, these studies provide additional support for the importance of the CA1 in spatial and verbal learning in adolescents as seen in the current study.

While we did observe trend-level associations between aerobic fitness and the right DG and CA4 subfields within the hippocampal head, these were no longer apparent after adjusting for covariates. Exercise induced neurogenesis within the DG in adolescent rat models specifically improves pattern separation abilities^84^, and ultimately contributes to superior spatial navigation abilities. Thus, it was surprising that the DG subfield volumes were not significantly associated with either aerobic fitness or spatial navigation abilities. It is very likely that aerobic exercise relates to cellular processes that may be less sensitive to detection via sMRI and/or lead to smaller effect sizes requiring larger sample sizes to detect. For example, while neurogenesis creates new granule cells, these new cells represent a very small number of the total cells within the DG^71^, and may not be detectable by utilizing sMRI. Nonetheless, the association between aerobic fitness and CA1 volumes detected in our sample may also be reflective of a potentially unique developmental profile of this subfield during adolescence. In a recent longitudinal sMRI study, the CA3, CA4, and DG were found to show small, steady decreases in volume from childhood through mid-adulthood^85^. The CA1, however, showed a quadratic pattern of development, with continued growth seen throughout late adolescence^85^. Regional specificity in the development of hippocampal subfield volumes may suggest that the CA1 is still undergoing macro-structural changes during adolescence, which may render it more susceptible to changes in neuroplasticity related to physical exercise that are observable by sMRI. In fact, while the DG is thought of as unique given its prolonged plasticity during adulthood; widespread plasticity is a hallmark of the adolescent brain^9,86^. In addition, previous human MRI studies that have observed both exercise and spatial memory induced changes in the DG focused on adult populations^32,34^. It is feasible that exercise may have larger effects in the DG in adulthood, whereas aerobic exercise may exert its effects on other hippocampal regions, such as the CA1, during this critical period of development. Given that gray matter differences across adolescence may result from a number of biological changes, including neuronal bodies, neuropil, glial cells, and capillaries—all of which may vary across hippocampal subfields and respond differentially to aerobic exercise—volumetric differences in relation to aerobic fitness in this current cross-sectional study require further investigation. For example, glial cells far outnumber neurons by a ratio of 50 to 1^87^, and exercise has been shown to increase cell density^22^ as well as remodel the morphology of astrocytes (i.e. a subclass of glial cells) within the medial temporal lobe in male rats^22,23^. Furthermore, beyond neurogenesis and neuropil, aerobic exercise may lead to neurovasculature changes that could contribute to larger gray matter volumes, as a recent exercise training study showed marginal associations of anterior hippocampal cerebral blood flow (CBF) with exercise in children^88^, and an intervention study in older adults showed that volumetric enlargement of the head region were accompanied by increases in CBF to the hippocampus^33^. Thus, additional longitudinal fitness and aerobic exercise intervention studies are needed in adolescent populations to test the plausible idea that the prolonged maturation of the CA1 region may make it more sensitive to the impact of exercise across adolescence. These future imaging studies should also employ a combination of structural morphometry, arterial spin labeling, and microstructural analyses, which may help to elucidate both macro and microstructural differences associated with exercise and learning ability.

Strengths and weaknesses of the study should be noted. First, the study design employed a spatial navigation memory task analogous to the rodent equivalent of the Morris Water Maze task, allowing for improved translation between human and animal studies. All youth were also of healthy weight, which is important given previous studies in children and adolescents have been unable to disentangle potential differences in the association between aerobic fitness and brain structure without the potential confounding factor of overweight individuals in the lesser fit groups^14,27^. In addition, the current study used methods to estimate subfield volumes in humans that are more likely to mirror subregional specificity delineations in animal models. However, it should be noted that methodological challenges inherent to hippocampal segmentation methods make it rather difficult to accurately demarcate the CA4 and DG regions, as the CA4 region includes the molecular-layer of the DG^26^. In addition, the CA3-4, DG, and head region delineation can be impacted by scan resolution and segmentation software^26^, and brain volumetric measurements can vary as a function of the age of the study population^89^. Thus, results of the current study should be treated as preliminary and interpreted with caution until replicated using higher resolution scan data. Additional limitations include that this study was correlational in nature and was conducted in a small sample of male participants recruited from the Pacific Northwest region of the United States. We chose to focus on males in the current study design to reduce variability given the notable sex differences that have been noted to impact the primary measurements of our study. Half of the male participants in our current study were also athletes, with 47% of the sample having VO_2_ peak (ml/kg/min) values falling above the 95th percentile based on their age and sex^90^. Thus, the current findings may not be generalizable to the general adolescent male population across the U.S. More research is needed to explore similarities and/or differences in larger, diverse samples including both sexes and adextrals. Furthermore, preliminary animal studies show that the neuroprotective effects of exercise during the juvenile period (e.g., cell proliferation number, dendritic arborization, BNDF and mTOR protein expression) are maintained after adolescence and throughout a sedentary adulthood^91^, suggesting that the impact of exercise during teenage years may have both unique and long-lasting protective effects well into adulthood. Thus, longitudinal and intervention studies are needed to more fully characterized how aerobic fitness may impact the developing brain throughout adolescence into adulthood.

In conclusion, the present study suggests CA1 subfield volume relates to aerobic fitness and spatial and verbal domains of learning performance during adolescence. The results shed light on how parallel animal and human studies may ultimately help to improve our understanding as to how exercise may relate to macroscale changes of the hippocampus. Further collaborations between experimental cellular neuroscience and human imaging studies are needed to better understand the neuroanatomical underpinnings of macrostructural changes at the voxel level.

## Methods

### Ethics statement

Acquisition of this dataset was approved by Institutional Review Board at Oregon Health & Science University. All procedures were performed in compliance with the Code of Ethics of the World Medical Association (Declaration of Helsinki). Written informed consent and assent were obtained from all youth and their parents/guardians, in accordance with the local Institutional Review Board regulations.

### Participants and study design

Details of this study design have been previously published^24,92–94^. Briefly, we enrolled 34 adolescent males ages 15 to 18 years. Written informed consent and assent were obtained from all youth and their parents/guardians, in accordance with the local Institutional Review Board regulations. All youth were administered a modified version of the Youth Adolescent Activity Questionnaire (YAAQ) to assess exercise participation over the year. The sample consisted of both athletes and non-athletes. Briefly, the YAAQ asks about participation in various types of physical activity (e.g., basketball, soccer, track, weightlifting, baseball, etc.) throughout the year, as well as the number of hours per week spent doing each activity. Youth were enrolled if they met “high” or “low” fit criteria. High-fit was defined as engaged in an average of ≥ 10 h per week of regular organized aerobic physical activity across one or more seasons within the past year. Low-fit youth were defined as individuals who had participated in ≤ 1.5 h of highly aerobic physical activity per week over the past year. High-fit youth were asked to participate in the study during the season in which they were most physically active based on their YAAQ self-report (see Supplementary Table S3 for aerobic fitness characteristics of participants from YAAQ self-report). Of the 34 participants enrolled, 17 were high-fit and 17 were low-fit. Although participants were initially enrolled in the study based on these self-reports, previous analyses using this sample found VO_2_ peak (ml/kg LBM/min) was a stronger predictor than group membership in relation to hippocampal volumes^95^. In addition, self-reports of aerobic training can be biased by perception^96^. Thus, the current study focused on objective measurement of VO_2_ peak as the primary independent variable of interest.

In designing the current study, we carefully considered and chose to limit various sample characteristics in order to reduce variability. For example, a number of intrinsic sex differences have been reported in aerobic fitness ability, overall physical fitness level, hippocampal volumetric, and virtual maze task differences^97–103^. As such, we begin to explore this question in the male population first, and specifically recruited one sex in order to reduce between sex variability within the study sample. Left handers were also excluded from the current study due to the demonstrated handedness (general handedness and sport-specific lateral preferences) on cerebral laterality during adolescence^104–106^. Additional exclusion criteria were a diagnosis of a DSM-IV psychiatric disorder, significant substance use (> 10 lifetime alcoholic drinks or 2 drinks/occasion, > 5 uses of marijuana, any other drug use, or > 4 cigarettes per day), reported history of psychotic disorders in biological parents, any major medical condition or significant head trauma, left-handedness^107^, or irremovable metal in the body. Eligible participants were asked to complete aerobic fitness testing, questionnaires, cognitive testing, and an MRI scan within a 1-week period. Given acute effects of exercise on cognition^108,109^, aerobic testing never preceded cognitive or MRI scanning assessments. To assess intellectual functioning, participants were administered the two-subtest version of the Weschler Abbreviated Scale of Intelligence^110^. Socioeconomic status information was gathered by administering the Hollingshead Index of Social Position questionnaire to parents^111^. Pubertal development during adolescence is a potential confound as physical exercise has been reported to delay pubertal maturation^112^. Thus, pubertal maturation was assessed for each subject using self-rating Pubertal Development Scale (PDS)^113^.

### Aerobic fitness assessment

Aerobic fitness was assessed by measuring peak oxygen consumption using the Bruce Protocol wherein participants ran on a graded treadmill at a speed of 1.7 mph and 10%, with increases in speed and grade every 3 min until volitional exhaustion^55^. Individual fitness levels were measured using VO_2_ peak, which is a measure of the highest rate of an individual’s body to transport and utilize oxygen during incremental exercise and is thought to be the gold standard of measurement for aerobic physical fitness^56^. Oxygen consumption was calculated using the exact same Vmax Series, V6200 Autobox computerized indirect calorimetry system for every participant. VO_2_ peak values were only considered valid if the participant had delivered maximal effort on a test, which can be defined as one of the following conditions^56,96,114–118^: (1) heart rate reaching 200 beats or greater per minute^96^, (2) a respiratory exchange ratio greater or equal to 1:0^96^, (3) or a plateau in oxygen consumption indicating a steady state despite an increased workload. These thresholds for a valid VO_2_ peak are based on previous findings in children and adolescents, which corroborate that maximum heart rate at VO_2_ peak is independent of age^96,119–121^, maturation^96,119,122,123^, and sex^96,124–126^ during this time of development. Furthermore, respiratory exchange ratios greater or equal to 1 following a progressive exercise test is an informative indicator of near maximal effort in adolescents^96^. Since body mass has been related to aerobic fitness performance and hippocampal volume, VO_2_ peak measurements were scaled by lean body mass (LBM) to calculate an objective measure of aerobic fitness as ml/kg LBM/min^127–132^. During the adolescent period, boys experience an increase in LBM, which accounts for robust changes in VO_2_ peak seen with age^133^. Thus, LBM based VO_2_ peak in ml/kg LBM/min was used in the current study to allow for a body mass independent measure of cardiopulmonary fitness^134^, and to reduce the possibility of body fat as a confounding variable.

While the intention of the study was to recruit both high and low-fit adolescents, in the current sample, all participants had a VO_2_ peak (ml/kg/min) in the top 50th percentile for their age and sex^90^. Likely owing to the inclusion of both athletes and non-athletes in the current study, 47% of the sample fell within the top 95th percentile. Seventeen youth qualified as “low-fit” on the YAAQ but displayed a high VO_2_ peak group average of 67 ml/kg LBM/min (see Supplementary Table S3). The discrepancy between low self-report measures of fitness activity and high VO_2_ peak average values may be due to inherent perception biases when self-reporting aerobic training^96^. Thus, given that VO_2_ peak is considered to be the “gold standard” of a single objective measurement of aerobic fitness, and previously analyses using this data have shown that LBM based VO_2_ peak (ml/kg LBM/min) was a stronger predictor than group membership in relation to hippocampal volumes^95^, the current study focused on LBM based VO_2_ peak as the primary independent variable of interest.

### Spatial and verbal memory assessment

Details of the spatial memory paradigm have been previously published using this dataset^24^. Briefly, spatial learning and memory were assessed using a computerized virtual Morris Water Maze video game (vMWT)^135^ (Fig. 1a). The virtual environment of the task consisted of a pool of water with a ceiling, floor, walls, and four objects placed around the pool. The practice trial consisted of a hidden platform in a pool, and participants were instructed to find the hidden platform as fast as possible. Using a joystick, participants “swam” over to the platform, and it emerged from the water 10s later. Following the practice trial, the participant completed 6 learning trials in 6 different learning environments to locate a hidden platform in another position. Each learning trial began from a different start position, and total distance traveled from each start position to the hidden platform was calculated for every trial, as well as total distance traveled within in each ‘quadrant’ of the pool. While the participant searched for and found the hidden platform, the computer recorded the x, y coordinate position approximately every 0.02s. Using this method, the computer calculated the total distance traveled in each quadrant of the pool, including the quadrant that the hidden platform was located in—known as the target quadrant. Percent distance traveled equaled the total distance traveled in the target quadrant divided by the total distance traveled across all quadrants. Percent distance traveled in the target quadrant was calculated for each of the 6 trials. Spatial learning across the 6 trials was measured as a metric of delta (δ); the change between percent distance traveled in the target quadrant between trial 1 and trial 6 (δ = trial 6 percent distance in target quadrant − trial 1 percent distance in target quadrant). 30 minutes after the completion of the learning trials on the vMWT, participants were asked to complete a spatial delay recall trial. The spatial delay recall trial was identical to the learning trials, but the hidden platform was removed. This was done to assess whether the participant remembered the location of the hidden platform that was identified during the previous 6 learning trials. Spatial delay recall was calculated as the percent distance traveled in the target quadrant within the first 30s of the trial.

Verbal memory was assessed by the Rey Auditory Verbal Learning Test (RAVLT)^136^ (Fig. 1b). A list of 15 words, called list A, were read consecutively for five trials (Trials A1-A5), and participants verbally listed as many words as possible after each presentation. A second 15-word list (list B) was read to the participant and they were then asked to recall the list (Trial B1). Participants were then asked to immediately recall words from list A (Trial A6). After a 20-minute delay, participants were asked to recall the words from list A (Trial A7). A verbal learning score was calculated by summing the total number of words learned from list A over 5 trials, and final scores were z-transformed. Delayed recall was calculated as the number words remembered from list A after a 20-min delay subtracted from words remembered from list A after the 5th trial (Trial A7–A1).

### MRI acquisition and image analysis

Images were acquired on a 3.0T Siemens Magnetom Tim Trio system (Siemens Medical Solutions, Erlangen, Germany) with a twelve-channel head coil at OHSU’s Advanced Imaging Research Center. Whole-brain, high-resolution structural anatomical images were acquired in the sagittal plane using a T_1_ weighted MPRAGE scanning sequence (TI = 900 ms, Flip Angle = 10°, TE = 3.58 ms, TR = 2300 ms, acquisition matrix = 256 × 240, resolution = 1 mm × 1 mm × 1.1 mm). Images were preprocessed using Freesurfer’s recon-all (v5.3, http://surfer.nmr.mgh.harvard.edu) processing pipeline which includes: removing non brain tissue, tissue segmentations of grey matter (GM), white matter (WM), CSF boundaries, and Talairach transformation in standard MNI space^137^. Intracranial volume (ICV) was calculated using Functional Magnetic Resonance Imaging of the Brain (FMRIB)’s automated segmentation tool (FAST) v4.1 to account for individual variability in brain size^138,139^.

Hippocampal subfields were calculated using FreeSurfer’s automated method in FreeSurfer v6.0 (beta version; http://surfer.nmr.mgh.harvard.edu)^26^. Prior publications have listed the technical details of the processing procedure^140,141^. This method provides hippocampal subfield volumetric measures that more closely align with histological measurements, compared to alternative automated segmentation algorithms and previous versions of the software^26,137,142^. Automated approaches have advantages over gold standard manual segmentation because of improved reliability^142^ and increase interstudy comparability. FreeSurfer segments the hippocampus in 12 regions of interest. These regions can be grouped as head, body, and tail (Fig. 2a). The head consists of the following subfields of interest: parasubiculum, presubiculum-head, subiculum-head, CA1-head, CA3-head, CA4-head, DG-head, molecular layer-head, and the hippocampal amygdala transition area (Fig. 2b). The body consists of the following subfields of interest: presubiculum-body, subiculum-body, CA1-body, CA3-body, CA4-body, DG-body, molecular layer body, and fimbria (Fig. 2c). Hippocampal images were created using Quantitative Imaging Toolkit (QIT)^143^.

### Statistical analysis

Data were analyzed using RStudio Version 1.1.463 and the following packages: reghelper, r2glmm, nlme, dplyr, and tidyr. We used a strategic model building approach to examine how the independent variables (IV) of aerobic fitness (i.e. VO_2_ peak), virtual Morris Water Maze performance (learning and memory), and verbal memory performance (learning and memory) related to subfield volume. Covariates ICV and PDS score were included in the model to account for individual variability in brain size, and the impact of pubertal development on hippocampal size^144–147^. Given the high correlation of subfield volumes between and within hemispheres for any given individual, linear mixed effects models were used to examine if there was an association between the independent variable (i.e. aerobic fitness, spatial learning, etc.) and volume as a function of the hippocampal subfield ROIs, with fixed effects for hemisphere, ICV, PDS, and subject as a random effect.

Based on the literature, an a priori model building strategy was used to examine both the anterior–posterior axis of the hippocampus, as well as subfield volumes based on the IV of interest (Fig. 2). First, we examined a potential interaction between the IV (i.e. VO_2_ peak, spatial learning, verbal learning, etc.) and regional differences in volume along the longitudinal axis (head, body, tail ROIs) of the hippocampus (Fig. 1a). This initial model for the IV of VO_2_ peak was:

$$\begin{aligned}&M1:\, {Volume}_{i}\\&\quad={b}_{0}+{b}_{1}\,{\mathrm{VO}2\mathrm{peak}}_{i}*{b}_{2}\,{LongAxis(Head,\,Body,\, Tail)}_{i}\\&\qquad+{b}_{3}{Hemisphere\, (Left,\, Right)}_{i}+{b}_{4}{ICV}_{i}+{b}_{5}{PDS}_{i} +{U}_{i}+{\varepsilon }_{i}\end{aligned}$$

Based on this initial result, we then completed one of two follow-up analyses to probe subfield volumes of interest. If the interaction of the initial model was significant, suggesting volume differences along the longitudinal axis (head, body, tail), follow-up analyses examined volume differences of the subfields of interest (i.e. CA1, CA3, CA4, DG, presubiculum, subiculum, and parasubiculum) within the longitudinal axis region identified by the initial model (Fig. 2b). This follow-up model was:

$$\begin{aligned}&M2: \,{Volume}_{i}\\&\quad={b}_{0}+{b}_{1}\,{\mathrm{VO}2\mathrm{peak}}_{i}*{b}_{2}\,{Head \,Specific\, Subfields(\mathrm{CA}1, \mathrm{CA}3, \mathrm{CA}4, \mathrm{DG})}_{i}\\&\qquad +{b}_{3}\,{Hemisphere \,(Left,\, Right)}_{i}+{b}_{4}{ICV}_{i}+{b}_{5}{PDS}_{i} +{U}_{i}+{\varepsilon }_{i}\end{aligned}$$

Specifically, if there was no support for differences in volume along the longitudinal axis (i.e. interaction term for the longitudinal axis was not significant), then the subfields of interest (i.e. CA1, CA3, CA4, DG, presubiculum, subiculum, and parasubiculum) were examined for the entire length of the hippocampal formation (Fig. 2c). For each IV, subfields were chosen a priori based on literature derived from previous animal^20,22^ and human subfield-related studies^58^. Model fits were then examined using the F-values and *p* values for the fixed effects via the anova.lme function. Given the number of regression analyses performed, Bonferroni corrections were applied to each group of tests per IV to reduce reporting Type I errors (2 primary models for aerobic fitness: *p* < 0.025; 4 primary models for vMWT and RAVLT: *p* < 0.0125), whereas to correct for the number of post-hoc follow-up analyses of these models to investigate up to 7 ROIs included a Bonferroni correction of *p* < 0.007. Together, this modeling strategy allowed for testing how aerobic fitness, spatial learning and memory, and verbal learning and memory relate to (1) volumetric differences along the anterior–posterior longitudinal axis of the hippocampus and (2) regional specificity of subfield volumes localized along this longitudinal axis. Finally, a mediation analysis outlined by Baron and Kinney^59^ was performed to test whether hippocampal subfield volumes would mediate the relationship between aerobic fitness and memory outcomes. All statistics reported are for the two-tailed test criterion.

## Supplementary Information

Supplementary Information.

## Data Availability

The datasets generated during and analyzed for the current study are available from the authors on reasonable request.
