# Supplementary material for: Understanding the role of aerobic fitness, spatial learning, and hippocampal subfields in adolescent males
Source: Sci Rep. 2021 Apr 29;11:9311. doi: 10.1038/s41598-021-88452-9 (PMC8084987; doi:10.1038/s41598-021-88452-9)
Supplement: Supplementary file 1 — Supplementary Information. [file 41598_2021_88452_MOESM1_ESM.docx]

**Supplementary Materials**

**Title: Understanding the Role of Aerobic Fitness, Spatial Learning, and Hippocampal Subfields in Adolescent Males**

Authors: Sandhya Prathap, Bonnie J. Nagel, and Megan M. Herting

**Supplementary Tables and Figures**

**Supplementary Table S1. Shapiro-Wilks test for fitness and brain volume variables.** The Shapiro-Wilks test investigates whether assumptions of normality are met for the relevant aerobic fitness and hippocampal subfield volume variables. Significant results suggest a deviation from normality.

|  | | |
| --- | --- | --- |
|  | **W** | **p** |
| *Aerobic fitness variables* | | |
| LBM based VO_2_ peak (ml/kg LBM/min) | 0.95 | 0.17 |
| VO_2_ peak (ml/kg/min) | 0.98 | 0.64 |
| *Subfield volume variables* |  |  |
| Left Whole Hippocampal Head | 0.97 | 0.55 |
| Right Whole Hippocampal Head | 0.98 | 0.88 |
| Left CA1 head | 0.96 | 0.30 |
| Right CA1 head | 0.99 | 0.99 |

**Supplementary Figure S2. Aerobic Fitness and Hippocampal Head Subfield Volume Associations.** Scatter plot demonstrates the linear model associations between aerobic fitness and **(a)** total hippocampus head volumes as well as subfield volumes within the head of the hippocampus, including **(b)** CA1 head, **(c)** DG head, **(d)** CA3 head, **(e)** CA4 head. N, adjusted R squares, beta coefficients, and p-values reflect a simple linear regression of aerobic fitness and hippocampal volume for each region of interest using the two tailed hypothesis criteria.


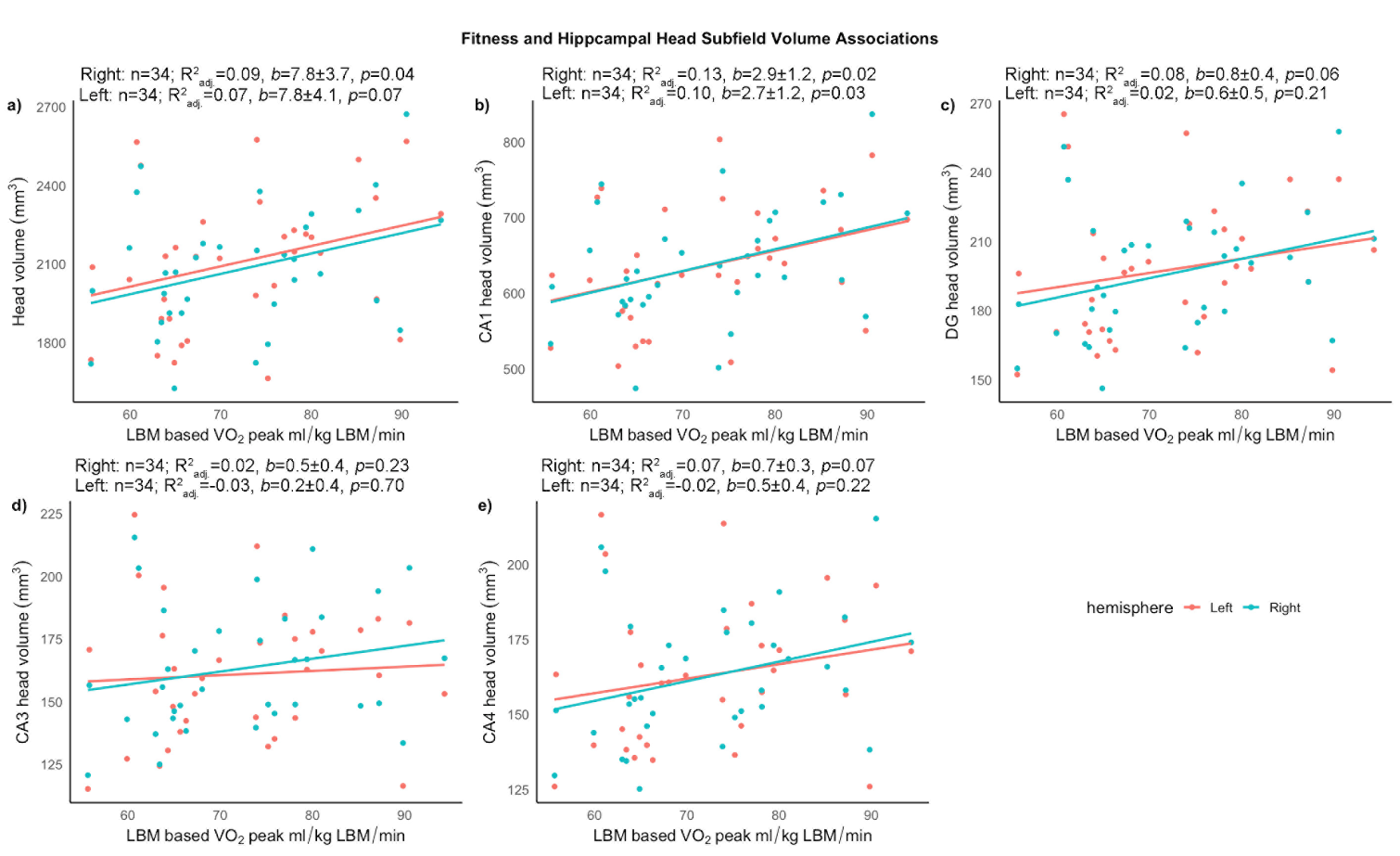


**Supplementary Table S3. Aerobic Fitness Variables Stratified by High and Low Fit groups.** Table demonstrates the mean and standard deviations for all aerobic fitness related variables including LBM based VO_2_ peak, an objective single measurement of aerobic fitness, as well as results from the Youth Adolescent Activity Questionnaire (YAAQ) which asks participants to self-report the number of hours of per week of aerobic activity over the past year, and within the season in which they had participated in the study.

|  | **High-Fit (n=17)** | **Low-Fit (n=17)** | **t-test** |
| --- | --- | --- | --- |
| LBM based VO_2_ peak (ml/kg LBM/min) | 77.7 (10.5) | 67.0 (7.4) | *t*(28.6) = -3.41, *p* = .002 |
| Aerobic activity (hr/week over past year) ^a^ | 11.3 (3.4) | .79 (1.5) | *t*(21.8) = -11.52, p < .001 |
| Aerobic activity (hr/week in season scanned) ^a^ | 12.6 (3.8) | 1.0 (1.8) | *t*(23.1) = -11.32, p < .001 |

a= Youth Adolescent Activity Questionnaire (YAAQ)
